# Nuchal Cystic Hygroma in Fetus: A Case Report

**DOI:** 10.7759/cureus.56018

**Published:** 2024-03-12

**Authors:** Esha Kohli, Anupama Sawal, Gaurav Kohli

**Affiliations:** 1 Research and Development, Jawaharlal Nehru Medical College, Datta Meghe Institute of Higher Education and Research, Wardha, IND; 2 Anatomy, Jawaharlal Nehru Medical College, Datta Meghe Institute of Higher Education and Research, Wardha, IND; 3 Radiology, Sudhir Diagnostics, Bhilai, IND

**Keywords:** lymphatic malformation, macrocystic, chromosomal aneuploidies, lymphangiomas, cystic hygromas

## Abstract

Cystic hygromas detected prenatally usually have a poor prognosis; hence, a correct and early diagnosis is essential. A prenatal ultrasound may detect a cystic hygroma as early as 10 weeks of gestation. Knowledge of the imaging findings and prognostic factors is necessary for effective perinatal counseling. Nuchal cystic hygromas (NCHs) in fetuses present a rare and challenging medical situation for prenatal care providers. This case report aims to describe a particular case of NCH detected through routine prenatal ultrasound, emphasizing the diagnostic demanding situations, management decisions, and final results. The etiology of NCHs remains multifactorial and complicated. Despite the fact that a few instances are sporadic, a great proportion has been associated with genetic aberrations, mainly chromosomal anomalies such as Turner syndrome, trisomy 21, and trisomy 18. Recent advances in molecular genetic testing, together with chromosomal microarray analysis and non-invasive prenatal testing, have facilitated the identification of the underlying genetic factors, contributing to a better knowledge of the pathogenesis of NCHs. In fetuses, they pose a complex scientific state of affairs with diverse implications. Advances in diagnostic techniques and genetic testing have notably progressed our capacity to become aware of related anomalies, offering precious insights into diagnosis and management alternatives. However, further research is warranted to get to the bottom of the underlying mechanisms of NCH development, enhance prenatal counseling, and refine therapeutic procedures to optimize outcomes for affected pregnancies.

## Introduction

Cystic hygromas, cystic lymphatic malformations, or macrocystic lymphatic malformations are a type of congenital lymphatic malformations [1], which can occur anywhere in the body but tend to occur mainly in the neck and axilla [2,3]. They are the most common subtypes of lymphangiomas. They arise from defective formation of neck lymphatics or failure of the lymphatic system to communicate with the venous system, i.e., failure of the jugular vein to communicate with the jugular lymphatic sac [4,5]. This leads to an accumulation of lymph and causes the formation of a cystic structure, most commonly in the cervical or axillary region. Determining the prevalence of nuchal cystic hygromas has been difficult because a large number may have been missed due to therapeutic termination of pregnancy. The prevalence has been estimated at 1/6,000 to 1/12,000 live births and 1/750 abortions. No separate figures are available for Indian populations. Cystic hygromas may be diagnosed on a prenatal ultrasound examination. They are seen as cystic structures, often located in the cervical region. The presence of a midline septum (nuchal ligament) within the cyst helps differentiate it from nuchal edema [1]. The color Doppler does not show any blood flow within the cyst. It is essential to correctly identify cystic hygromas and look for associated findings such as hydrops fetalis, cardiac malformations, and skeletal abnormalities [6]. Cystic hygromas are frequently diagnosed in the first trimester and affect about one in every 285 pregnancies. About 50%-80% can be associated with chromosomal aneuploidies. Turner’s syndrome is the most common aneuploidic association followed by trisomy 21 and trisomy 13 [1]. The patient was advised to undergo amniocentesis to rule out aneuploidy; however, the patient refused and elected to terminate the pregnancy elsewhere.

## Case presentation

**Figure 1:**
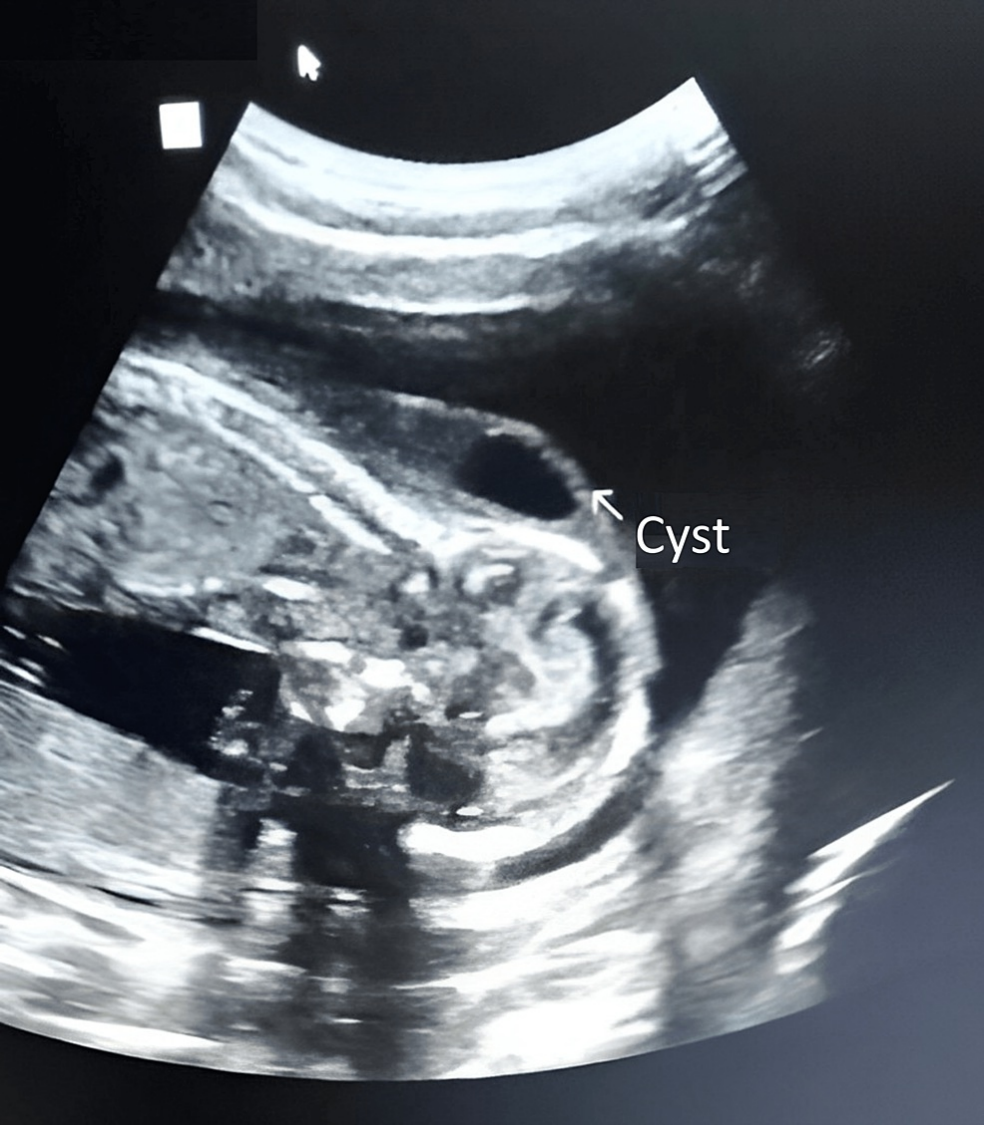
Longitudinal view showing the septate nuchal cystic hygroma.

**Figure 2:**
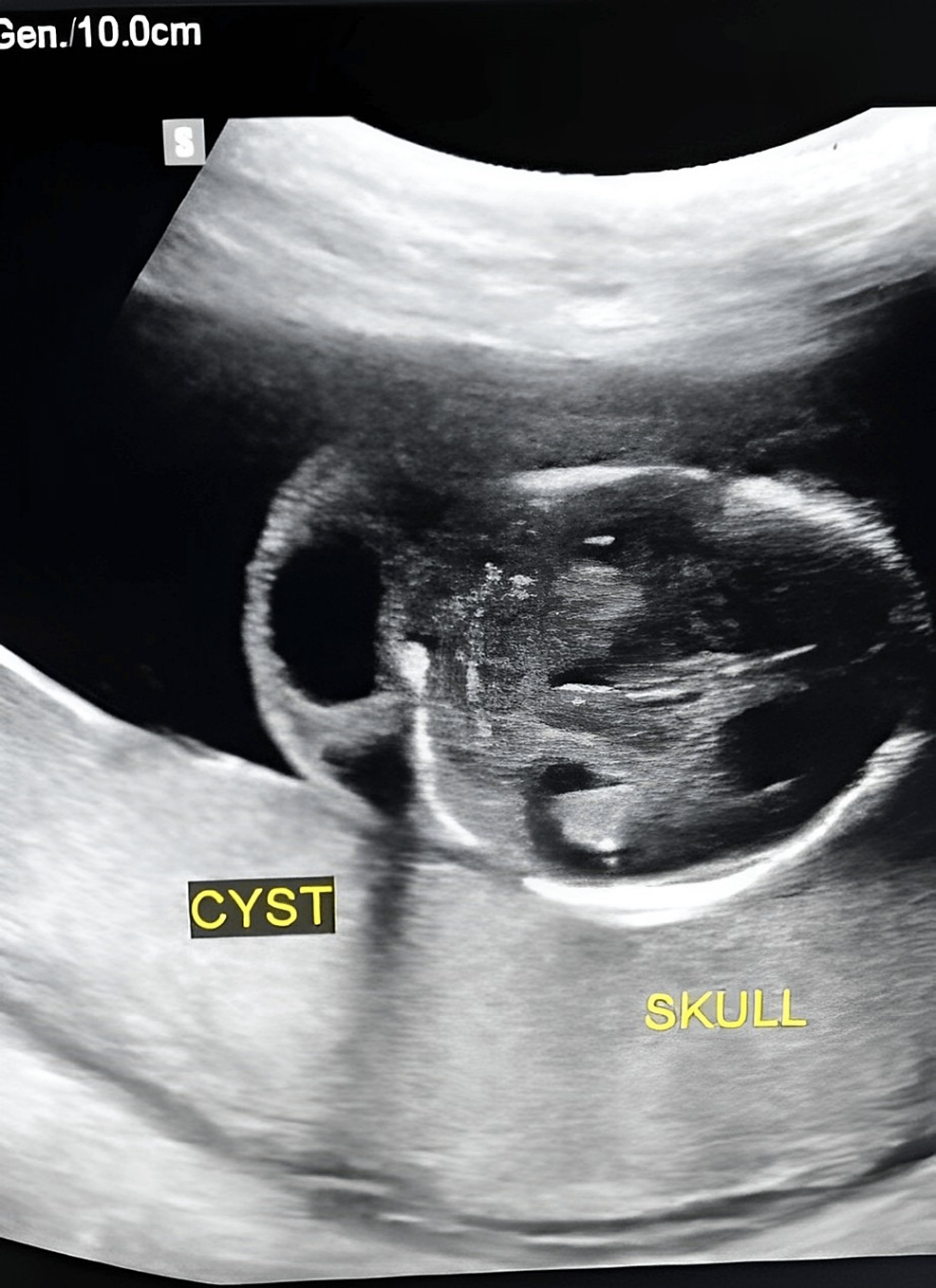
Transverse view showing the septate nuchal cystic hygroma.

**Figure 3:**
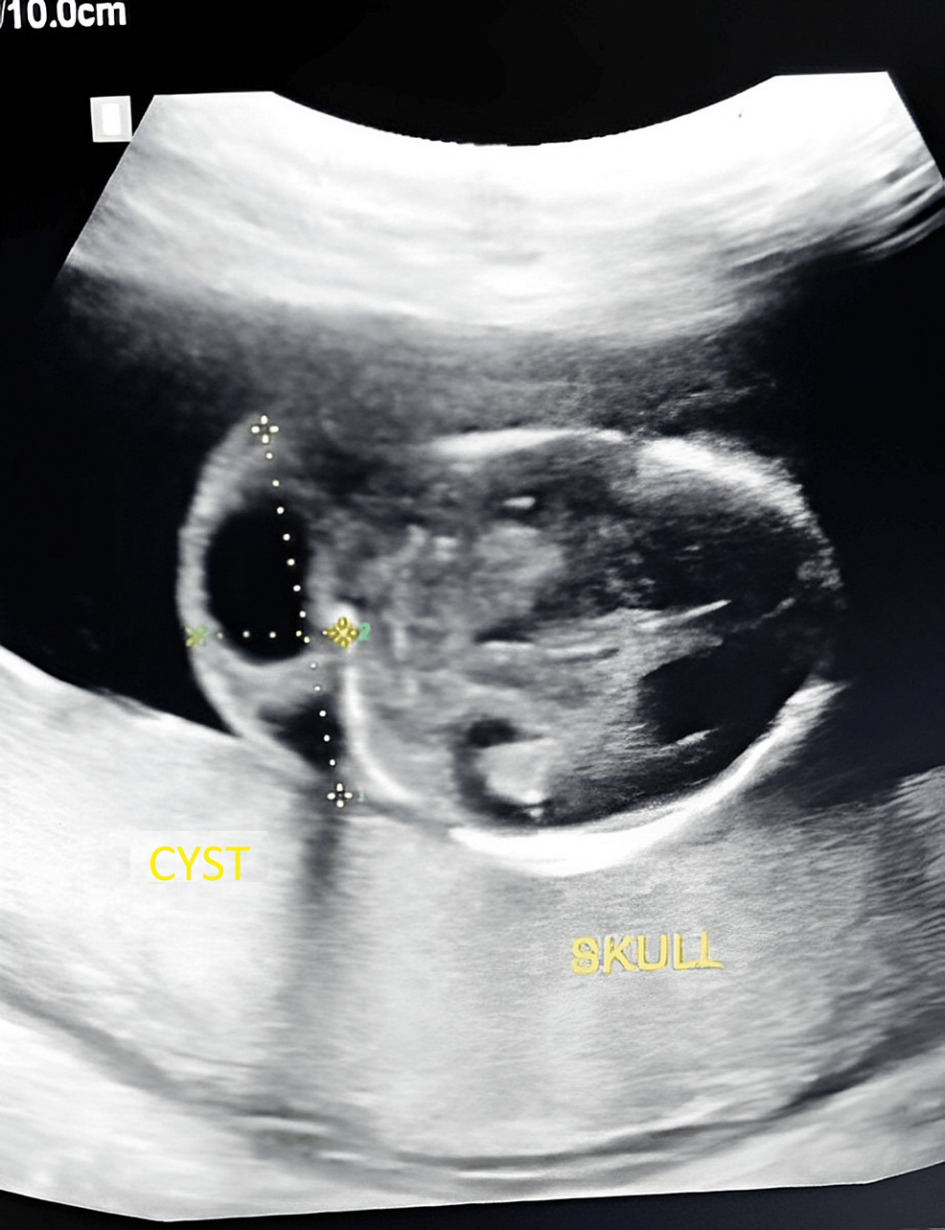
Transverse view showing hygroma having thickness of 12.8 mm. Thickness of nuchal cystic hygromas greater than 6 mm is associated with poor prognosis; in the above figure, thickness (value 2) is 12.8 mm; so, this case has poor prognosis. Value 1 has no such relevance and is not significant for the given case.

**Figure 4:**
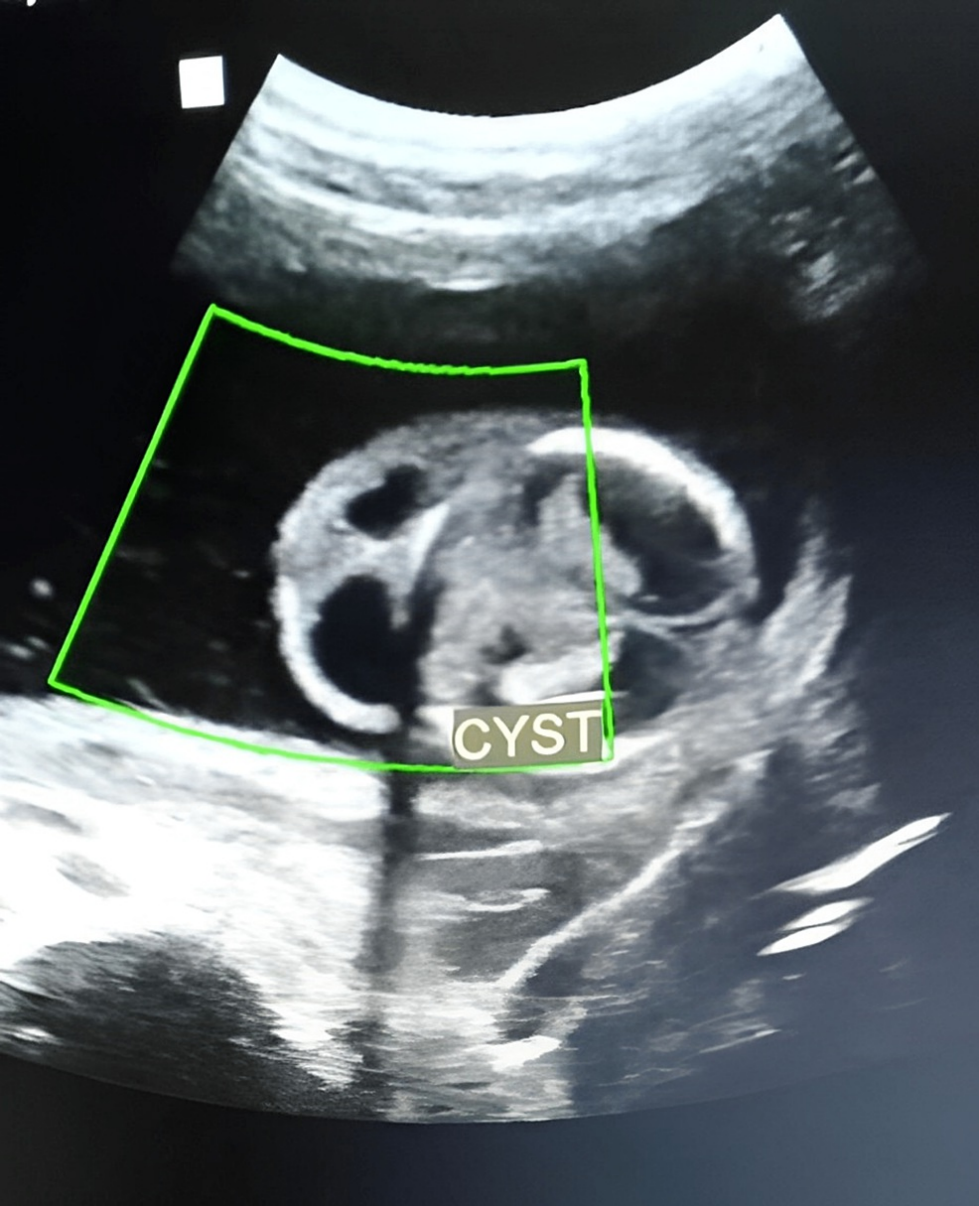
Color Doppler sonography does not show any flow in the lesion.

A 25-year-old pregnant female (G2P1) with natural conception came for routine obstetric ultrasonography (USG). She had been married for six years. Her gestational age was 16 weeks and four days. She denied any history of surgery or previous major illnesses. There was no history of any exposure to teratogenic substances. She had a vegetarian diet, and there is no history of smoking or alcohol intake. She was referred for routine obstetric sonography to find out the condition of the fetus and fetal growth. At home, her urinary pregnancy test was positive, and she had not undergone any previous sonography or blood tests during this pregnancy. She had one full-term normal delivery and one spontaneous abortion at seven weeks of gestational age. A transabdominal sonography of the gravid uterus was performed. This demonstrated a single live intrauterine fetus of 16 weeks and five days duration. There was evidence of a cystic mass in the fetal neck with a single internal septation (Figures 1, 2). The cyst has a maximum thickness of 12.8 mm measured from an intact skull at the transverse view (Figure 3). No skull defect could be seen. Color Doppler did not show any flow in the lesion (Figure 4). No other significant finding was present. The parents refused genetic testing and elected to terminate the pregnancy elsewhere.

## Discussion

This is a case of nuchal cystic hygroma in a 16-week, four-day-old fetus. Cystic hygromas are lymphatic malformations in which cavernous lymphatic spaces communicate and develop to form huge cysts that can infiltrate the encircling tissues. Cystic hygromas are macrocystis, i.e., lymphatic structures greater than 2 cm. Microscopically, they are lined by endothelium with scanty stroma. There is an association between cystic hygromas and chromosomal aneuploidy, fetal hydrops, and intrauterine fetal death. About half of fetal cystic hygromas have chromosomal aneuploidies [7,8]. The most common abnormalities are Turner’s syndrome and Down’s syndrome followed by trisomy 13 and triploidy [9]. Fetal nuchal cystic hygromas can usually be detected on prenatal ultrasound. Elevated alpha-fetoprotein levels between 15 and 20 weeks can be seen. Prenatal USG findings of nuchal cystic hygromas include a thin-walled intradermal cystic region in the cervical region. According to Rosati and Guariglia [8], nuchal cystic hygromas are an area of echolucency in the soft tissue of the occipital region consisting of two symmetric cavities completely separated by the midline septum without or with internal trabeculae. Cystic hygromas have poor outcomes due to their association with chromosomal abnormalities, hydrops fetalis, or intrauterine fetal demise. Unfavorable outcome is as high as 77.8%. Several factors can influence the outcome of cystic hygromas [7]. Prenatal management consists of serial USG follow-up, MRI, or intrauterine injections of sclerosing agents. Cystic hygromas in the anterior neck are prone to airway obstruction, and ex utero intrapartum treatment is indicated. For patients with a normal karyotype and favorable analysis, further sessions with a pediatric healthcare professional are crucial. A study suggests that patients without chromosomal or structural abnormalities may be treated by intrauterine sclerosing agents [10,11]. Poor prognostic factors are fetal hydrops, thickness of cystic hygroma greater than 6 mm, and septated cystic hygromas, in association with other major malformations. Similar cases of nuchal cystic hygromas greater than 6 mm thickness have associated chromosomal aneuploidy, and poor fetal outcome is as high as 90%. Cases with a thickness of less than 6 mm and no associated aneuploidy may resolve spontaneously.

## Conclusions

While doing an antenatal ultrasound exam, careful evaluation of the fetal neck region is of the utmost importance. Nuchal cystic hygromas must be considered as a differential in any abnormalities of the cervical region. Additional views and further evaluation must be done. Familiarity with these conditions and associated findings is key to the diagnosis and management. In summary, our case reports nuchal cystic hygroma in the fetus, which is a benign lymphatic malformation. Recognition of this condition is important as most of the cases have unfavorable outcomes, and assessment of the prognostic factors is important for proper patient counseling. In this case, the prognosis was explained to the patient, and she was advised of genetic testing; however, she elected to terminate the pregnancy at a higher center. No follow-up was available.
